# GenCPM: a toolbox for generalized connectome-based predictive modeling

**DOI:** 10.3389/fnins.2025.1627497

**Published:** 2025-09-29

**Authors:** Baijia Xu, Shengxian Ding, Wanwan Xu, Carolyn Fredericks, Yize Zhao

**Affiliations:** ^1^Department of Biostatistics and Bioinformatics, Rollins School of Public Health, Emory University, Atlanta, GA, United States; ^2^Department of Biostatistics, Yale School of Public Health, Yale University, New Haven, CT, United States; ^3^Department of Neurology, Yale School of Medicine, Yale University, New Haven, CT, United States

**Keywords:** brain connectome, generalized linear model, regularization, survival analysis, Alzheimer's disease

## Abstract

Understanding brain—behavior relationships and predicting cognitive and clinical outcomes from neuromarkers are central tasks in neuroscience. Connectome-based Predictive Modeling (CPM) has been widely adopted to predict behavioral traits from brain connectivity data; however, existing implementations are largely restricted to continuous outcomes, often overlook essential non-imaging covariates, and are difficult to apply in clinical or disease cohort settings. To address these limitations, we present GenCPM, a generalized CPM framework implemented in open-source R software. GenCPM extends traditional CPM by supporting binary, categorical, and time-to-event outcomes and allows the integration of covariates such as demographic and genetic information, thereby improving predictive accuracy and interpretability. To handle high-dimensional data, GenCPM incorporates marginal screening and regularized regression techniques, including LASSO, ridge, and elastic net, for efficient selection of informative brain connections. We demonstrate the utility of GenCPM through analyses of the Anti-Amyloid Treatment in Asymptomatic Alzheimer's Disease (A4) Study and the Alzheimer's Disease Neuroimaging Initiative (ADNI), showing enhanced predictive performance and improved signal attribution compared to standard methods. GenCPM offers a flexible, scalable, and interpretable solution for predictive modeling in brain connectivity research, supporting broader applications in cognitive and clinical neuroscience.

## 1 Introduction

Advancements in brain mapping technologies, such as diffusion magnetic resonance imaging (MRI), functional MRI (fMRI), and electroencephalography (EEG), have significantly expanded our ability to study brain connectivity or the connectome. These techniques provide rich temporal and spatial data, allowing researchers to construct connectivity maps that capture the intricate functional and structural relationships between different brain regions. Functional connectivity (FC), mostly derived from fMRI time series data, captures statistical dependencies between different brain nodes and serves as an essential marker for studying individual differences in cognition and mental health (Van Den Heuvel and Pol, 2010). Structural connectivity (SC), typically derived from diffusion MRI, maps the anatomical pathways of white matter tracts that physically link brain regions (Zhang et al., 2022), and provides complementary insight by revealing the underlying architecture that constrains and supports functional interactions.

The Connectome-based Predictive Modeling (CPM) framework (Shen et al., 2017) is a widely used machine learning framework to predict behavioral and cognitive outcomes from FC or SC data. In brief, CPM extracts individual edges from brain connectivity matrices to serve as input features and employs penalized regression techniques such as ridge regression (Gao et al., 2019), within a cross-validation framework to identify informative brain connections and construct predictive models of brain–behavior relationships. This approach has been successfully applied across a variety of studies (Greene et al., 2018; Finn et al., 2015; Rosenberg et al., 2016), particularly when the target outcomes are continuous behavioral traits such as fluid intelligence. Recent advances have sought to improve CPM's interpretability and generalization. For instance, (Gao et al. 2018) introduced a multidimensional CPM (mCPM) framework that integrates connectivity matrices from various task states using canonical correlation analysis to capture richer brain-behavior associations. Similarly, (Yang et al. 2023) developed a modular CPM approach that leverages both task-based and resting-state fMRI to forecast working memory performance, emphasizing modularity and predictive generalizability. These studies highlight the field's ongoing efforts to extend CPM's applicability and biological interpretability, goals that align closely with the development of GenCPM.

Despite its success, CPM has two key limitations that restrict its applicability. First, most existing CPM implementations focus exclusively on continuous outcomes (e.g., task performance, symptom severity), limiting their feasibility to broader neurobiological and clinical research questions. In clinical or disease cohort studies, primary outcomes of interest often include diagnostic status, disease progression, or time to symptom onset, etc. These outcome traits are inherently categorical or time-to-event variables, for which conventional CPM frameworks are not equipped to handle. For instance, in the study of neurodegenerative disorders like Alzheimer's Disease (AD), the ability to predict time-to-conversion from mild cognitive impairment (MCI) to AD is crucial. Approximately 10%–15% of individuals with MCI develop AD each year (Rosenberg and Lyketsos, 2008), and survival analysis has been widely used to model disease progression and mortality risk (Todd et al., 2013). However, current brain connectome-based predictive studies have not fully integrated connectivity data with survival analysis, limiting our scope to characterize early and accurate predictions about disease onset and progression. Second, existing CPM frameworks do not explicitly incorporate non-imaging covariates, such as demographic factors, genetic variants, and socioeconomic status, despite growing evidence suggesting that these variables significantly influence brain-behavior relationships (Ossenkoppele et al., 2020; Cuyvers and Sleegers, 2016; Karp et al., 2004). While some studies aim to isolate the unique contribution of brain connectivity, neglecting non-imaging confounders and population heterogeneity may lead to biased estimates and misleading conclusions. Notably, in many mental health and neurological studies, brain connectivity features may contribute only marginally to outcome prediction once clinical and demographic variables are considered. Moreover, interactions among non-imaging covariates can be crucial for identifying meaningful subgroups and enhancing model interpretability. Ignoring these factors within the CPM framework can substantially diminish both predictive performance and scientific insight.

To address these limitations, we propose an enhanced predictive modeling framework, Generalized Connectome-based Predictive Modeling (GenCPM), that extends CPM in several key directions. First, we introduce a flexible modeling pipeline that accommodates binary, categorical, and time-to-event outcomes, extending the applicability of CPM beyond continuous variables. This is particularly valuable for disease cohort studies, including characterizing progression in neurodegenerative diseases such as AD. Additionally, our approach allows for the incorporation of user-specified non-imaging covariates (e.g., demographic, genetic, and socioeconomic factors) alongside connectivity data, thereby improving predictive performance and interpretability. Analogous to the original CPM framework, GenCPM includes a marginal screening that filters irrelevant features before model fitting. To induce sparsity while retaining key predictive signals, this is followed by penalized regression techniques, including Least Absolute Shrinkage and Selection Operator (LASSO) (Tibshirani, 1996), ridge regression (Hoerl and Kennard, 1970), and the elastic net (Zou and Hastie, 2005). Penalized regression methods are well suited for high-dimensional datasets where the number of predictors often exceeds the number of observations. By adding a penalty term to the loss function, these methods constrain model complexity and prevent overfitting, promoting sparsity or shrinkage in the selected features and thereby enhancing both predictive accuracy and model interpretability (Hastie et al., 2015). Within the CPM framework, such regularization facilitates robust edge selection and improves generalizability by identifying the most predictive connectome among thousands of potential edges in the brain network. By integrating these enhancements, our approach provides a more versatile and robust framework for studying brain-behavior relationships. Notably, our GenCPM toolbox available to be downloaded at http://github.com/BXU69/GenCPM is implemented in R, a freely available and open-source statistical software platform, offering a cost-effective and accessible alternative to those implemented in MATLAB, which requires a paid license. Finally, to demonstrate the utility and flexibility of the GenCPM toolbox, we apply it to achieve connectivity-based prediction under two large AD neuroimaging datasets: the Anti-Amyloid Treatment in Asymptomatic Alzheimer's Disease (A4) study and the Alzheimer's Disease Neuroimaging Initiative (ADNI) study. These applications demonstrate GenCPM's improved performance in predicting a range of outcomes, including continuous cognitive measures, binary diagnostic classifications, categorical memory scores, and time-to-event data for amyloid positivity conversion.

The rest of the paper is organized as follows. In Section 2, we present the GenCPM framework in detail, including the underlying statistical models, marginal and regularized feature selection strategies, and the design and implementation of the toolbox. In Section 3, we showcase the predictive performance and feature selection results obtained from applications to the A4 and ADNI datasets. Finally, Section 4 discusses the implications of our findings and outlines potential future directions.

## 2 Materials and methods

### 2.1 Toolbox overview

**Figure 1:**
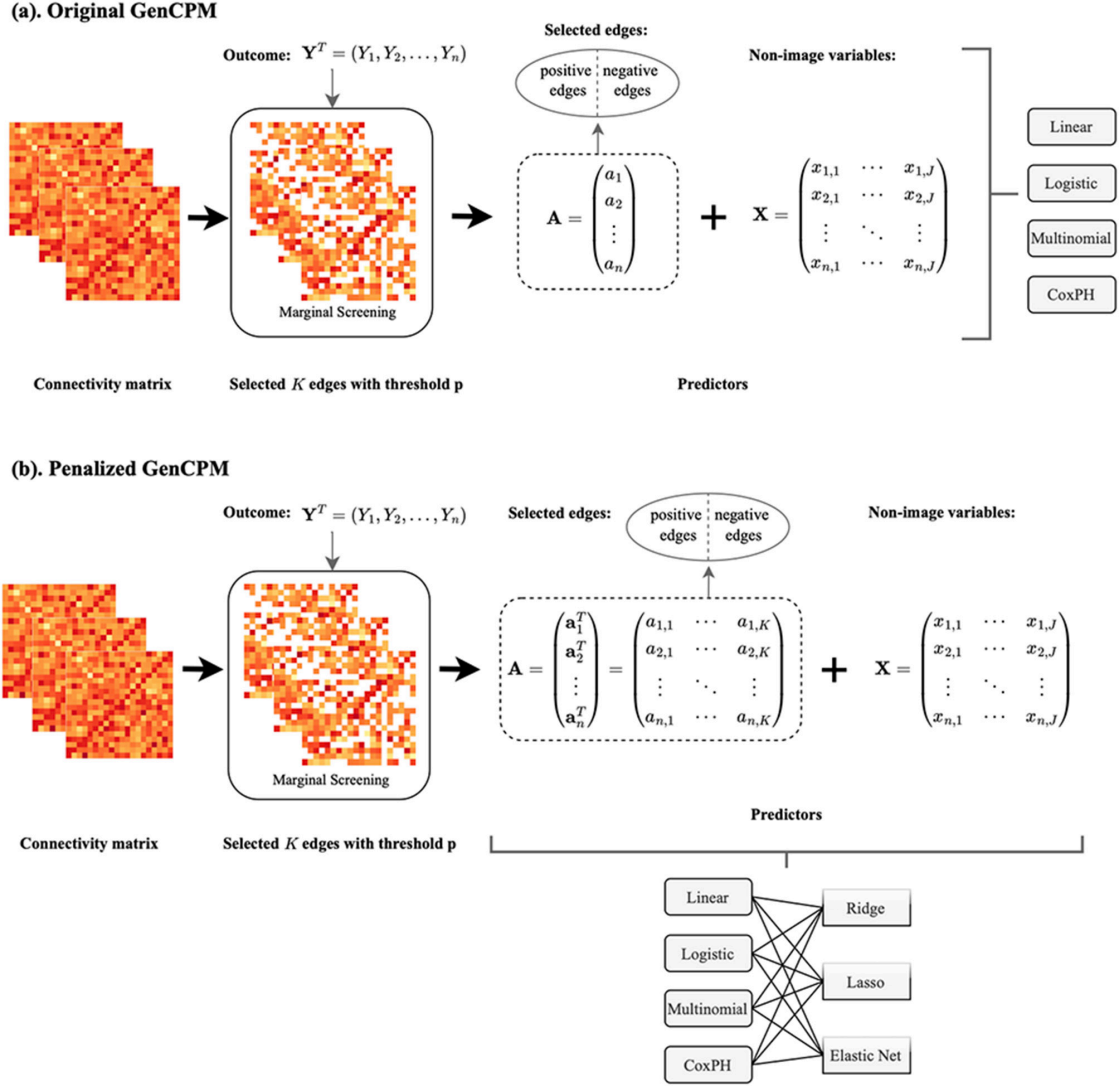
Overview of the GenCPM toolbox. Each subject provides a connectivity matrix and an outcome variable (e.g., a behavior or clinical measure). **(a)** In the original GenCPM framework, marginal screening is applied to select the top *K* significant edges based on a predefined threshold *p*. The selected edges are separated into positively and negatively correlated sets, and summary measures, computed as sums of the average connectivity strength within each set, are derived for each subject. These connectivity-derived predictors are then combined with optional non-imaging covariates and entered into downstream models, including linear, logistic, multinomial, and CoxPH regression. **(b)** In the penalized GenCPM variant, the full set of selected edge features is retained without aggregation, allowing the model to capture fine-grained individual edge-level contributions. The resulting feature matrix, combined with non-imaging covariates, is then input into regularized regression models such as LASSO, ridge, and elastic net, where penalization is applied only to edge features, for joint modeling and feature selection while preserving the contribution of non-imaging covariates.

Figure 1 illustrates the overall workflow of the proposed GenCPM toolbox pipeline, consisting of two version, i.e. original GenCPM and penalized GenCPM. For each subject, a connectivity matrix generated from SC or FC and the behavioral or clinical outcome of interest are input into the pipeline. In the first stage, marginal screening is performed to select the *K* most significant edges based on a threshold *p* of a statistical metric, e.g. *p-*value. In the original GenCPM pipeline (Figure 1a), a scalar summary *a*_*i*_ is computed for each subject, forming an edge predictor ***A*** ∈ ℝ^*n*^. At this step, users can choose between two options for edge predictor construction: (1) summarizing all selected edges, without distinguishing positive or negative correlations, into a single scalar predictor per subject; (2) categorizing selected edges into positively or negatively correlated sets with the outcome, and summarizing each set separately for two models. The resulting edge predictor(s) are then combined with non-imaging variables ***X***, which may include demographic or clinical features such as age, gender, and genetic information (e.g., APOE genotype). Depending on the type of outcome variable, different statistical models are applied, including linear regression, logistic regression, multinomial regression, or Cox proportional hazards (CoxPH) regression, using the combined set of edge-based and non-imaging predictors. In the penalized GenCPM version (Figure 1b), rather than computing a scalar summary, the full set of selected edge-level features is retained for each subject, constructing a richer predictor matrix ***A*** ∈ ℝ^*n*×*K*^. As in the original GenCPM pipeline, users can either fit a combined model using all selected edges or fit separate models for the positive and negative sets. After integration with non-imaging features ***X***, penalized regression models are applied, including LASSO, ridge, and elastic net. LASSO and elastic net perform secondary screening, enabling edge selection, whereas ridge regression shrinks coefficients without eliminating any edges. Notably, secondary screening by regularization is applied exclusively to the edges, without filtering the non-imaging features. In other words, all non-imaging features are maintained in the model fitting process.

Additionally, GenCPM offers an evaluation function to assess the model fitting accuracy as well as a function to visualize the selected edges at a large-scale brain network level through heatmaps. The default map offered is based on the Shen268 atlas (Shen et al., 2017), to 10 functional networks: 1. Medial frontal, 2. Frontoparietal, 3. Default mode, 4. Motor, 5. Visual I, 6. Visual II, 7. Visual association, 8. Limbic, 9. Barsal ganglia, and 10. Cerebellum (Greene et al., 2018).

### 2.2 Marginal screening

Marginal screening is a feature selection technique used to reduce the dimensionality of datasets by evaluating each feature individually based on its marginal association with the response variable. This approach involves calculating a specific statistic (e.g., correlation coefficient, *p-*value, t-statistic, etc.) for each feature to determine its relevance to the outcome variable. Features that meet a predefined threshold for this statistic are retained for further analysis, while those that do not meet the threshold are discarded. Marginal screening is particularly useful in high-dimensional datasets where the number of features far exceeds the number of observations, as it helps to eliminate irrelevant or redundant variables early in the modeling process, thereby simplifying the model and reducing computational complexity.

In this study, the marginal screening test is conducted by correlating each edge with the behavior outcome and the most significantly correlated edges are selected by a predefined threshold of *p*. For continuous, binary, and categorical outcomes, *p* is the cut-off value for the p-value in Spearman correlation. In the context of survival data, marginal screening can be adapted to identify important edges that are associated with time-to-event outcomes. Marginal screening in survival analysis often employs techniques such as the Cox proportional hazard model or log-rank tests to evaluate the relationship between each feature and the survival time. In our implementation, for time-to-event outcomes, marginal screening is conducted using the Cox Proportional Hazards regression model, where the p-value from the univariate analysis serves as the screening threshold. Edges exhibiting a strong marginal association with the survival outcome are selected for inclusion in more complex multivariate models.

### 2.3 Model construction

#### 2.3.1 Generalized linear model for continuous, binary and categorical outcome

During CPM, edges (i.e., correlations between nodes in a FC or SC matrix) are correlated with outcome variables in a training dataset to identify predictive networks. Single-subject summary statistics are computed as the sum of edge weights in each network and are used to generate linear predictions, which are subsequently transformed into binary or categorical outcomes via the appropriate link functions. The resultant coefficients are applied to new subjects' connectivity matrices (test data) to produce independent sample predictions, which are again transformed into discrete outcomes. A Generalized Linear Model (GLM) is a flexible generalization of ordinary linear regression that allows the response variable to follow error distributions beyond the normal distribution, such as binomial or multinomial distributions (Nelder and Wedderburn, 1972). Formally, let the behavior outcome vector be denoted by $y={\left(y_{1},y_{2},...,y_{n}\right)}^{T}$, assumed to be a realization of a random variable $Y={\left(Y_{1},...,Y_{n}\right)}^{T}$ whose components are independently distributed with means $\mu ={\left(\mu_{1},...,\mu_{n}\right)}^{T}$. Let ***A*** ∈ ℝ^*n*×*K*^ represent the matrix of selected edge measures after marginal screening, where each row corresponds to a subject and each column to a selected edge, and let ***X*** ∈ ℝ^*n*×*J*^ denote the matrix of non-imaging covariates. We define the combined predictor matrix as X^*^ = (***A***, ***X***) ∈ ℝ^*n*×(*K*+*J*)^, integrating selected edges and non-imaging features across all *n* subjects.

To generalize from ordinal linear models to generalized linear models, we provide the following three-part specification (McCullagh, 2019):

1. **Random component**: Each component of ***Y*** is assumed to follow a distribution from the exponential family, with a density function of the form

$$\begin{array}{l} Y_{i}\sim f\left(y_{i};\theta_{i},\phi \right)=exp\left\{\frac{y_{i}\theta_{i}-b\left(\theta_{i}\right)}{a\left(\phi \right)}+c\left(y_{i},\phi \right)\right\} \end{array}$$

where θ_*i*_ is the canonical parameter, ϕ is the dispersion parameter, and *a*(·), *b*(·), *c*(·) are known functions that characterize the distribution.

2. **Systematic component**: A linear predictor $\eta ={\left(\eta_{1},...,\eta_{n}\right)}^{T}$ is defined as

$$\begin{array}{l} \eta_{i}=\overset{K+J}{\underset{j=1}{\sum }}\beta_{j}x_{ij}^{*}=x_{i}^{*T}\beta \end{array}$$

where $x_{i}^{*}={\left(x_{i1}^{*},...,x_{i\left(K+J\right)}^{*}\right)}^{T}$ denotes the covariate vector for subject *i* from X^*^, and $\beta ={\left(\beta_{1},...,\beta_{K+J}\right)}^{T}$ are unknown parameters to be estimated.

3. **Link function**: A link between the random and systematic components is established through:

$$\begin{array}{l} \eta_{i}=g\left(\mu_{i}\right)=g\left(E\left(Y_{i}\right)\right)=x_{i}^{*T}\beta , \end{array}$$

where *g*(·) is a monotonic and differentiable link function.

Therefore, generalized linear models extend ordinal linear models by allowing the mean **μ** and the linear predictor **η** to be related through an arbitrary link function. In ordinary linear models, the identity link is used, making **η** and **μ** identical and suitable for outcomes distributed over the entire real line (Nelder and Wedderburn, 1972). In logistic regression, a GLM designed for binary outcomes, the canonical link function is the logit function:


$$\begin{array}{l} \eta_{i}=\log\left(\frac{p_{i}}{1-p_{i}}\right), \end{array}$$

where *p*_*i*_ denotes the probability that the binary response equals 1. For multinomial logistic regression, which handles response variables with more than two categories, the logit link is extended:


$$\begin{array}{l} \eta_{i}=\log\left(\frac{\pi_{ij}}{\pi_{ij^{*}}}\right), \end{array}$$

where π_*ij*_ is the probability that subject *i* belongs to category *j*, and $\pi_{ij^{*}}$ is the probability of reference category j^*^. This extension allows the model to accommodate multi-class response variables.

By supporting non-normal distributions for the response variables and by incorporating flexible link functions, GLMs extend the applicability of linear models to a wider range of outcome types. Such a framework is widely used for accurately modeling the relationship between predictors and non-normally distributed outcomes, which makes it suitable for a broad range of applications (Agresti, 2015).

#### 2.3.2 CoxPH model for time-to-event outcome

The CoxPH model (Cox, 1972), also known as the Cox regression, is a principal statistical technique for analyzing censored survival data and assessing the influence of multiple variables on the time until an event occurs. It is the most widely used regression model for time-to-event data due to its ability to model survival outcomes without requiring specification of the baseline hazard function. In recent years, the CoxPH model has proven to be a reliable and efficient tool to analyze disease cohorts including those in AD research (Spooner et al., 2020). For instance, in the context of our data applications, the survival time *T* is defined as the time from a subject's first clinical visit to either the date of *Aβ* positivity diagnosis (for AD converters), or the last follow-up date (for censored subjects). Subjects who received an *Aβ* pathology diagnosis are considered AD converters, labeled with δ_*i*_ = 1, while those without a diagnosis during the study period are treated as censored, labeled with δ_*i*_ = 0. The CoxPH model specifies the hazard function for subject *i* at time *t* as the product of an unspecified baseline hazard *h*_0_(*t*) and an exponential function of the subject's covariates. In our framework, the covariates include both the selected edges ***A*** and the non-imaging covariates ***X***, combined into a full predictor matrix X^*^.

Following the assumptions in (Tibshirani 1997), we assume no tied event times, that is, no two subjects convert from *Aβ*-negativity to *Aβ*-positivity at exactly the same number of days. Additionally, the CoxPH model assumes proportional hazards: the hazard ratio at time *t* to the baseline hazard remains constant over time. Assuming that the effect of covariates on the hazard is constant over time, the Cox model specifies the hazard function for subject *i* at time *t* as


$$\begin{array}{l} \left[h\left(t|x_{i}^{*}\right)=h_{0}\left(t\right)\exp\left(x_{i}^{*T}\beta \right),\right] \end{array}$$

where $X^{*}={\left(x_{1}^{*},...,x_{n}^{*}\right)}^{T}\in {\mathbb{R}}^{n\times \left(K+J\right)}$ represents the combined predictor matrix, and $\beta ={\left(\beta_{1},...,\beta_{K+J}\right)}^{T}$ are the unknown model parameters to be estimated. Therefore, the hazard for an individual is modeled as the product of two terms: *h*_0_(·), the baseline hazard common to all individuals, and *exp*(·), the hazard ratio that captures multiplicatively how much higher or lower that individual's hazard will be when compared to the baseline level, according to an individual's covariates.

Given that the baseline hazard *h*_*i*_(·) is unspecified, (Cox 1972) proposed estimating **β** through partial likelihood maximization, defined as


$$\begin{array}{l} \left[L\left(\beta \right)=\underset{k\in D}{\prod }\frac{exp\left(x_{k}^{*T}\beta \right)}{\sum_{j\in R_{k}}exp\left(x_{j}^{*T}\beta \right)},\right] \end{array}$$

where *D* denotes the set of indices corresponding to observed events (δ_*k*_ = 1), and *R*_*k*_ is the risk set at time *t*_*k*_, consisting of all individuals still at risk immediately prior to *t*_*k*_.

### 2.4 Regularization methods in secondary screening

Our toolbox provides three penalized regression techniques, including LASSO, ridge, and elastic net, to improve model stability and predictive performance. Among these, only LASSO and elastic net are applicable for secondary edge selection following the preliminary marginal screening, as ridge regression does not perform variable selection and thus cannot be used for this purpose. Importantly, penalization is applied exclusively to the screening of edge features, while all non-imaging variables are retained as fixed covariates during model fitting. These regularization techniques can be applied in conjunction with various model types – linear, logistic, multinomial, and CoxPH regression – depending on the nature of the response variable.

Considering the GLM introduced in Section 2.3.1 and the CoxPH model in Section 2.3.2, let $Y={\left(Y_{1},...,Y_{n}\right)}^{T}\in {\mathbb{R}}^{n}$ denote the outcome vector and X^*^ = (***A***, ***X***) ∈ ℝ^*n*×(*K*+*J*)^ denote the full predictor matrix, where ***A*** ∈ ℝ^*n*×*K*^ contains selected edge measures after first screening and ***X*** ∈ ℝ^*n*×*J*^ contains non-imaging covariates. The coefficient vector is denoted by $\beta ={\left(\beta_{A}^{T},\beta_{X}^{T}\right)}^{T}\in {\mathbb{R}}^{K\times J}$, where $\beta_{A}\in {\mathbb{R}}^{K}$ corresponds to edge features and $\beta_{X}\in {\mathbb{R}}^{J}$ corresponds to non-imaging variables. By adding an L1-penalty to the log likelihood function *l*(**β**; ***Y***, X^*^) for the GLM or the log partial likelihood for the CoxPH model (Tibshirani, 1996, 1997), the LASSO estimate of **β** is obtained by


$$\begin{array}{l} {\hat{\beta }}_{LASSO}=\underset{\beta }{\text{argmin}}\left\{-l\left(\beta ;Y,X^{*}\right)+\text{λ}||\beta_{A}{||}_{1}\right\}. \end{array}$$

The L1-penalty not only shrinks the coefficients toward zero but also sets some of them to exactly zero, thereby performing variable selection. This enables the identification of the most significant predictors, making the resulting model simpler and more interpretable, particularly in high-dimensional settings.

Unlike LASSO, ridge regression (Hoerl and Kennard, 1970) applies an L2-penalty term, which shrinks the coefficients toward zero without setting any exactly to zero, effectively reducing the variance of coefficients and improving the prediction accuracy. The ridge estimator is given by


$$\begin{array}{l} {\hat{\beta }}_{ridge}=\underset{\beta }{\text{argmin}}\left\{-l\left(\beta ;Y,X^{*}\right)+\text{λ}||\beta_{A}{||}_{2}^{2}\right\}. \end{array}$$

Although ridge regression does not perform variable selection, it is especially useful when the number of predictors exceeds the number of observations or when multicollinearity among predictors is present, as it stabilizes coefficient estimates and improves prediction accuracy.

Elastic net regression, proposed by (Zou and Hastie 2005), combines both L1 and L2 penalties. The elastic net estimator minimizes


$$\begin{array}{l} {\hat{\beta }}_{EN}=\underset{\beta }{\text{argmin}}\left\{-l\left(\beta ;Y,X^{*}\right)+{\text{λ}}_{1}||\beta_{A}{||}_{1}+{\text{λ}}_{2}||\beta_{A}{||}_{2}^{2}\right\}. \end{array}$$

Alternatively, introducing α = λ_2_/(λ_1_ + λ_2_), the elastic net optimization can be rewritten as


$$\begin{array}{l} {\hat{\beta }}_{EN}=\underset{\beta }{\text{argmin}}\left\{-l\left(\beta ;Y,X^{*}\right)\right\},\\ \text{subject to}\left(1-\alpha \right)||\beta_{A}{||}_{1}+\alpha ||\beta_{A}{||}_{2}^{2}\leq t, \end{array}$$

for some constant *t*. When α = 1, the penalty reduces to the LASSO penalty; when α = 0, it reduces to the ridge penalty (Hastie et al., 2015). This hybrid approach allows the elastic net to perform both variable selection and coefficient shrinkage simultaneously. By balancing the strengths of LASSO and ridge, elastic net offers a flexible and powerful method, particularly suited for scenarios involving highly correlated predictors or when the number of features exceeds the number of observations. It often achieves better generalization performance compared to either LASSO or ridge regression alone (Zou and Hastie, 2005).

### 2.5 Model evaluation

When fitting predictive models, it is essential to validate model performance and assess the quality of model fitting. Cross-validation helps mitigate overfitting and provides a more generalizable estimate of predictive performance by averaging evaluation metrics across multiple data partitions. Model evaluation using cross-validation (Stone, 1974) involves splitting the dataset into subsets to systematically assess a model's predictive accuracy and generalizability. The GenCPM toolbox provides two cross-validation options: *K*-fold cross-validation and leave-one-out cross-validation (LOOCV). In *K*-fold cross-validation, the dataset is divided into *K* approximately equal folds. The model is trained on *K*−1 folds and tested on the remaining fold, repeating this process *K* times so that each fold serves as a test set once. The resulting performance metrics are then averaged across folds to provide a robust estimate of model performance. Alternatively, LOOCV leaves out one observation at a time for testing while training the model on the remaining *n*−1 observations. Although LOOCV is computationally expensive, it is especially useful for small datasets, offering nearly unbiased performance estimates. In addition to internal cross-validation procedures, GenCPM also supports external validation using an independent dataset when available. Users can supply an *external connectome* and corresponding covariates through optional arguments (*external.connectome* and *external.x*), enabling the model to generate predictions and evaluate performance on unseen data. This allows for more robust generalization assessment beyond cross-validation. When these inputs are not provided, GenCPM defaults to internal *K*-fold or LOOCV as described above.

The evaluation metrics used depend on the model types and the prediction task. For linear regression models, the mean squared error (MSE) and the Pearson correlation coefficient are commonly used. MSE measures the average squared difference between observed and predicted values, with lower values indicating better predictive accuracy. Pearson correlation, on the other hand, quantifies the strength of the linear association between predicted and observed outcomes, with higher values indicating better alignment in trend and directionality. Together, these metrics provide a comprehensive assessment of model performance. For logistic regression models, performance is typically evaluated using the area under the receiver operating characteristic curve (AUC) (Hanley and McNeil, 1982), which quantifies the model's ability to discriminate between binary classes. An AUC of 1 indicates perfect classification, whereas an AUC of 0.5 reflects performance equivalent to random guessing. Thus, higher AUC values indicate better classification performance. For multinomial regression models, which address categorical outcomes with more than two classes, the AUC can be generalized to multinomial AUC using a one-vs-rest approach, where binary AUCs are computed for each class separately. In survival analysis, where the outcomes are time-to-event data, model performance is assessed using the concordance index (C-index) (Harrell et al., 1982). The C-index measures the concordance between predicted and observed event times by evaluating whether, for a randomly selected pair of individuals, the subject with the higher predicted risk indeed experiences the event earlier. The C-index ranges from 0.5 (no better than random) to 1.0 (perfect concordance), with higher values indicating better discriminative ability.

### 2.6 GenCPM design and implementation

#### 2.6.1 Package structure and functionalities

The GenCPM package consists of three primary modules: (1). *Original GenCPM*, (2). *Penalized GenCPM*, and (3). *Evaluation and Visualization*. Table 1 provides a comprehensive overview of the functions associated with each module, along with their descriptions. The *Original GenCPM* and *Penalized GenCPM* modules each include four functions designed to fit different types of generalized models: linear regression, logistic regression, multinomial regression, and Cox proportional hazards regression. The *Evaluation and Visualization* module comprises functions for model performance assessment and visualization of significant networks identified by the pipeline through heatmaps.

**Table 1:** Main functions implemented in GenCPM package.

**Function**	**Description**
**Original GenCPM**	
linear.GenCPM	Connectomes are used as linear predictors for a continuous outcome.
logit.GenCPM	Logistic regression for a binary-type outcome.
multinom.GenCPM	If the outcome is categorical, this function implements multinomial logistic regression.
cox.GenCPM	Generalized connectome-based survival modeling with time-to-event outcome using Cox regression.
**Penalized GenCPM**	
linear.regularized.GenCPM	Connectomes are used as linear predictors for a continuous outcome with additional regularization (LASSO, ridge, or elastic net penalty).
logit.regularized.GenCPM	Logistic regression for a binary-type outcome with regularization on connectivity edges.
multinomial.regularized.GenCPM	Multinomial regression for a categorical outcome with regularization.
cox.regularized.GenCPM	Generalized connectome based survival modeling with time-to-event outcome adding regularization.
**Evaluation and visualization**	
assess.GenCPM	Assessing the model performance across testing folds with varying types of metrics based on the specific model type.
heatmap.GenCPM	Visualize the selected edges either from thresholding or regularization based on the 10-node network label in Shen268 atlas.

Table 2 outlines the input variables, customizable arguments, and output variables for both *Original GenCPM* and *Penalized GenCPM* functions. Inputs include the connectivity matrices, outcome variables, and optional covariates. Arguments allow users to specify key model parameters, such as the type of cross-validation, penalty method, edge usage strategy, and regularization hyperparameters. Outputs summarize the selected edges, predicted outcomes, and statistical measures such as correlation coefficients and p-values.

**Table 2:** Variables and descriptions of original GenCPM and penalized GenCPM functions.

**Variable**	**Description**
**Input**	
connectome	Input connectivity array for model fitting, of dimension *M*(edges) × *M*(edges) × *N*(obs).
behavior	Response variable vector. Continuous for linear.GenCPM and linear.regularized.GenCPM.
	A factor with two levels for logit.GenCPM and logit.regularized.GenCPM.
	A more-than-two-level factor for multinom.GenCPM and multinom.regularized.GenCPM.
	Not applicable for cox.GenCPM and cox.regularized.GenCPM.
x	Non-imaging covariates matrix for model fitting, of dimension *n*(obs) × *p*(vars).
external.connectome	External connectivity array for prediction, of dimension *M*(edges) × *M* (edges) × *N*(obs).
external.x	External non-imaging covariate matrix for prediction, of dimension *n*(obs) × *p*(vars).
time	The follow-up time. Only applicable for cox.GenCPM and cox.regularized.GenCPM.
status	The status indicator, normally 0 = alive, 1 = dead. Only applicable for cox.GenCPM and cox.regularized.GenCPM.
**Argument**	
cv	Type of cross-validation. The default is leave-one-out CV. “k-fold” for K-fold CV.
k	The number of folds. The default is the sample size (leave-one-out CV).
correlation	The method for finding the correlation between edge and behavior. The default is “pearson.” Alternative approaches are “spearman” and “kendall.”
thresh	Threshold used for selecting significant edges. The default is 0.01.
edge	Usage of edges to fit models. “separate” for fitting two separate models using positive correlated edges and negative correlated edges respectively,
	and “combined” for fitting only one model using all edges selected. The default is “separate.”
type	Type of penalty. “lasso” for LASSO, “ridge” for ridge, and “EN” for elastic net. The default is “lasso.”
lambda	A user-specified lambda sequence or the optimal one automatically searched by *cv.glmnet*.
alpha	The elastic net mixing parameter, ranging from 0 to 1. The default is 0.95.
seed	A single value to specify a seed when generating training and test sets. The default is 1220.
**Output**	
positive_edges	All selected edges having a significantly positive relationship with behavior response.
negative_edges	All selected edges having a significantly negative relationship with behavior response.
r_mat	A list of matrices consisting of correlation coefficient between edges and behavior.
	Not applicable for cox.GenCPM and all .regularized.GenCPM functions.
p_mat	A list of matrices consisting of p-value from correlation between edges and behavior.
	Not applicable for cox.GenCPM and all .regularized.GenCPM functions.
positive_model	Fitted model using positive edges. Only applicable when users input external.connectome.
negative_model	Fitted model using negative edges. Only applicable when users input external.connectome.
combined_model	Fitted model using both positive and negative edges. Only applicable when users input external.connectome.
positive_predicted_behavior	Predicted behaviors from the positive model. Not applicable for cox.GenCPM and cox.regularized.GenCPM.
negative_predicted_behavior	Predicted behaviors from the negative model. Not applicable for cox.GenCPM and cox.regularized.GenCPM.
predicted_behavior	Predicted behaviors from the combined model. Not applicable for cox.GenCPM and cox.regularized.GenCPM.
positive_predicted_linear_predictor	Predicted linear predictors from the positive Cox regression model.
	Only applicable for cox.GenCPM and cox.regularized.GenCPM.
negative_predicted_linear_predictor	Predicted linear predictors from the negative Cox regression model.
	Only applicable for cox.GenCPM and cox.regularized.GenCPM.
predicted_linear_predictor	Predicted linear predictors from the combined Cox regression model.
	Only applicable for cox.GenCPM and cox.regularized.GenCPM.
actual_behavior	Actual values of behavior response. Not applicable for cox.GenCPM and cox.regularized.GenCPM.
actual_time	Actual values of survival time. Only applicable for cox.GenCPM and cox.regularized.GenCPM.
actual_status	Actual values of status indicator. Only applicable for cox.GenCPM and cox.regularized.GenCPM.
positive_lambda_total	The final lambda indicating penalty used in the positive model for each fold during cross-validation.
	Only applicable for .regularized.GenCPM functions.
negative_lambda_total	The final lambda indicating penalty used in the negative model for each fold during cross-validation.
	Only applicable for .regularized.GenCPM functions.
lambda_total	The final lambda indicating penalty used in the combined model for each fold during cross-validation.
	Only applicable for .regularized.GenCPM functions.

Similarly, Tables 3, 4 organize the key components associated with the *assess.GenCPM* and *heatmap.GenCPM* functions respectively. Table 3 details the inputs, arguments, and outputs for the assess.GenCPM function. The inputs include the GenCPM object returned by either *Original GenCPM* or *Penalized GenCPM* modules. The arguments specify model types and edge usage settings, while the outputs provide a range of evaluation metrics, including Pearson correlation coefficients (r), MSE, *p*-values, and AUC. Table 4 focuses on the *heatmap.GenCPM* function, listing its required inputs: the GenCPM object and a user-specified fold threshold parameter, which defines the selection criteria for edges based on their frequency of selection across cross-validation. The *heatmap.GenCPM* function produces a heatmap visualizing connectivity patterns between the 10 functional networks defined by the Shen268 atlas. In the visualization, red and blue colors represent positive and negative edges, respectively, with color intensity reflecting the degree of significance for each network.

**Table 3:** Variables and descriptions of assess.GenCPM function.

**Variable**	**Description**
**Input**	
object	Returned GenCPM object from one of .GenCPM or .regularized.GenCPM functions.
**Argument**	
model	A character string representing one of the built-in regression models.
	“linear” for linear.GenCPM and linear.regularized.GenCPM; “logistic” for logit.GenCPM and logit.regularized.GenCPM;
	“multinom” for multinom.GenCPM and multinom.regularized.GenCPM;
	and “cox” for cox.GenCPM and cox.regularized.GenCPM. The default is “linear”.
edge	Usage of edges to fit models. “separate” for fitting two separate models using positive edges and negative edges respectively,
	and “combined” for fitting only one model use all edges selected. The default is “separate”.
**Output**	
positive_r	Pearson correlation coefficient between predicted values from the input model and actual values.
	Only applicable for positive models fitted by linear.GenCPM and linear.regularized.GenCPM.
negative_r	Pearson correlation coefficient between predicted values from the input model and actual values.
	Only applicable for negative models fitted by linear.GenCPM and linear.regularized.GenCPM.
r	Pearson correlation coefficient between predicted values from the input model and actual values.
	Only applicable for combined models fitted by linear.GenCPM and linear.regularized.GenCPM.
positive_MSE	Mean Square Error of predicted values from the input model.
	Only applicable for positive models fitted by linear.GenCPM and linear.regularized.GenCPM.
negative_MSE	Mean Square Error of predicted values from the input model.
	Only applicable for negative models fitted by linear.GenCPM and linear.regularized.GenCPM.
MSE	Mean Square Error of predicted values from the input model.
	Only applicable for combined models fitted by linear.GenCPM and linear.regularized.GenCPM.
positive_AUC	Area Under Curve (AUC) values for logistic and multinomial logistic models.
	Only applicable for positive models fitted by logit. and multinom. functions.
negative_AUC	Area Under Curve (AUC) values for logistic and multinomial logistic models.
	Only applicable for negative models fitted by logit. and multinom. functions.
AUC	Area Under Curve (AUC) values for logistic and multinomial logistic models.
	Only applicable for combined models fitted by logit. and multinom. functions.
positive_cindex	Concordance index evaluated for the Cox regression model.
	Only applicable for positive models fitted by cox.GenCPM and cox.regularized.GenCPM.
negative_cindex	Concordance index evaluated for the Cox regression model.
	Only applicable for negative models fitted by cox.GenCPM and cox.regularized.GenCPM.
cindex	Concordance index evaluated for the Cox regression model.
	Only applicable for combined models fitted by cox.GenCPM and cox.regularized.GenCPM.
positive_predicted_behavior	Predicted behaviors from positive models. Not applicable for cox.GenCPM and cox.regularized.GenCPM.
negative_predicted_behavior	Predicted behaviors from negative models. Not applicable for cox.GenCPM and cox.regularized.GenCPM.
predicted_behavior	Predicted behaviors from combined models. Not applicable for cox.GenCPM and cox.regularized.GenCPM.
positive_predicted_linear_predictor	Predicted linear predictors from positive Cox regression models. Only applicable for cox.GenCPM and cox.regularized.GenCPM.
negative_predicted_linear_predictor	Predicted linear predictors from negative Cox regression models. Only applicable for cox.GenCPM and cox.regularized.GenCPM.
predicted_linear_predictor	Predicted linear predictors from combined Cox regression models. Only applicable for cox.GenCPM and cox.regularized.GenCPM.
actual_behavior	Observed values of behavior response. Not applicable for cox.GenCPM and cox.regularized.GenCPM.
actual_time	Observed values of survival time. Only applicable for cox.GenCPM and cox.regularized.GenCPM.
actual_status	Observed values of status indicator. Only applicable for cox.GenCPM and cox.regularized.GenCPM.

**Table 4:** Variable and description of heatmap.GenCPM function.

**Input**	**Description**
object	A GenCPM object output from one of the generalized regression model functions (linear.GenCPM, logit.regularized.GenCPM, etc.)
foldThreshold	Edges selected for over this many folds will be plotted.
	For example, if “foldThreshold” is set to be 1, then all selected edges across all folds are plotted.
	If it is set as 0.5, edges selected at least half of the time are plotted. The default is 0.5.

#### 2.6.2 Implementation example in R

**Figure 2:**
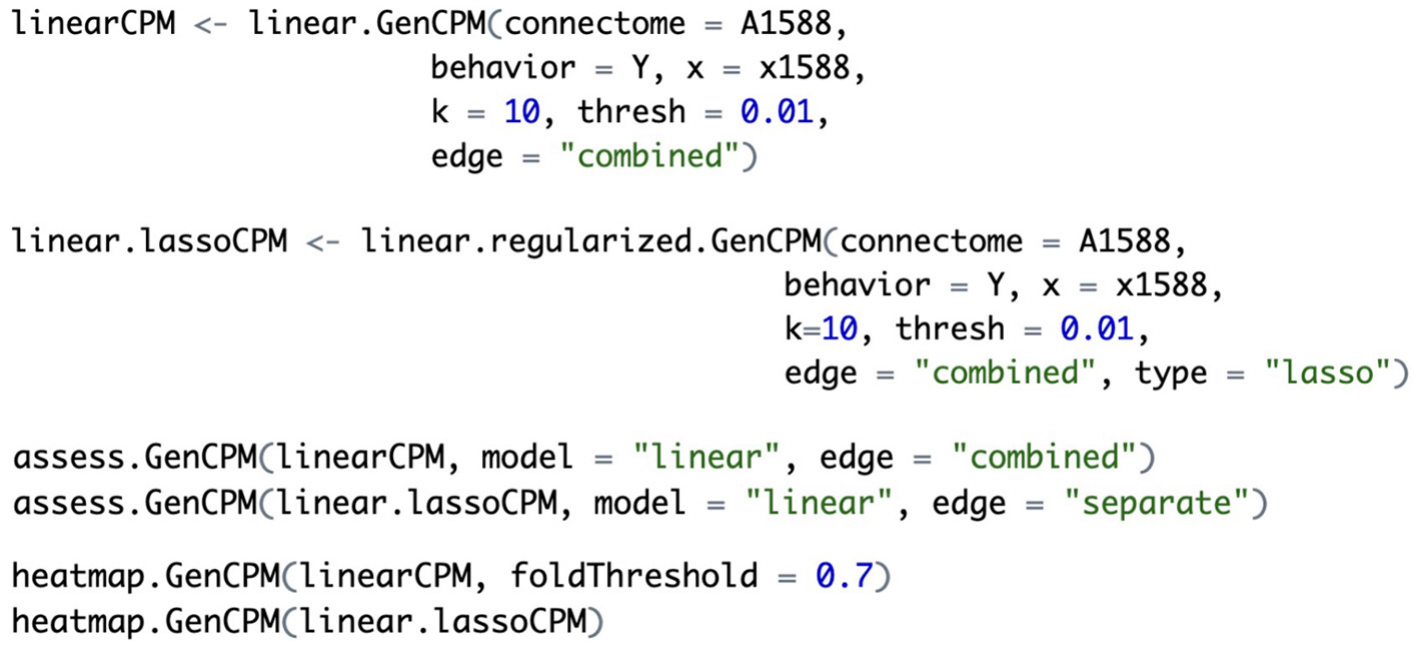
Examples of GenCPM implementation in R.

To illustrate the usage of the GenCPM toolbox, Figure 2 presents a code example demonstrating model construction, evaluation, and visualization. First, a linear GenCPM model is fitted using the *linear.GenCPM* function, requiring the input connectivity array (“A1588”), behavioral response variable (“Y”), and non-imaging covariate matrix (“x1588”). Key parameters include k = 10 (10-fold cross-validation), thresh = 0.01 (edge selection threshold), and edge = “combined” (combining positive and negative edges). Penalized linear modeling is conducted using *linear.regularized.GenCPM* function, specifying the type = “lasso” parameter to apply LASSO regularization. Model performance is then evaluated with *assess.GenCPM* function. It is critical to ensure that model type and edge usage match the input object returned by the *linear.GenCPM* or *linear.regularized.GenCPM* function to avoid evaluation errors. Finally, the *heatmap.GenCPM* function visualizes significant edges selected across folds, mapped onto 10 functional networks. The foldThreshold parameter controls which edges are visualized based on their selection frequency and defaults to 0.5. For example, setting “foldThreshold = 0.7” visualizes only those edges selected in at least 70% of folds.

### 2.7 Datasets for toolbox demonstration

To demonstrate the utility and flexibility of GenCPM, we apply the toolbox to two independent neuroimaging AD cohorts. The A4 study is used to showcase generalized linear models for continuous, binary, and categorical outcomes based on FC; and the ADNI study is used to demonstrate survival analysis with Cox proportional hazards modeling based on SC.

#### 2.7.1 A4 study: application for generalized linear model

We demonstrate the generalized modeling functions on the preclinical cohort from the A4 Study, a major clinical trial aiming at preventing AD by targeting cognitively normal older adults (ages 65–85) with elevated amyloid plaque levels. The study assesses whether reducing the amyloid burden can delay or prevent cognitive decline. To construct FC for each subject, T1-weighted (T1w) MRI scans are skull stripped using optiBET and nonlinearly aligned to the MNI-152 template using BioImage Suite (BIS). Functional MRI scans are preprocessed through slice timing correction and motion correction using SPM12, followed by motion scrubbing (mean frame-to-frame displacement [FFD] > 0.3 mm). Nuisance regression is performed to remove confounds using a 24-parameter motion model and signals from cerebrospinal fluid, white matter, and global signal. Data are smoothed using a 6 mm full-width at half-maximum (FWHM) Gaussian kernel and normalized to standard space. FC matrices are constructed by computing the Pearson correlation between the mean time series of each pair of regions defined by the Shen268 functional atlas.

The final dataset includes 1,588 subjects (954 females, 634 males), with a mean age of 71.54 years and an average of 16.62 years of education. Non-imaging covariates incorporated into the analysis include age, gender, years of education, and APOE4 carrier status (Safieh et al., 2019). Inclusion of APOE4 enables exploration of how biomarker trajectories prior to pathology onset may differ by genetic risk group. We apply GenCPM to model three types of outcomes: (1) **Continuous outcome**: The amyloid composition score, used in the linear regression model, reflecting the belief of underlying AD pathology driven by β-amyloid (*Aβ*) production and deposition (Murphy and LeVine III, 2010); (2) **Binary outcome**: Elevated amyloid status, defined by thresholding the amyloid composition score at a predefined cutoff, and modeled using logistic regression; (3) **Categorical outcome**: Immediate recall total scores from a memory test, binned into five discrete classes (0–10, 10–12, 12–14, 14–16, and 16–25) (Helkala et al., 1988), used as the outcome in multinomial logistic regression to reflect memory performance levels. While the immediate recall score was treated as a nominal variable and modeled using multinomial logistic regression as a demonstration of our toolbox's functionality, we acknowledge that the variable should more appropriately be treated as ordinal. In future analyses, we may extend the toolbox to include ordinal logistic regression, which would be a more suitable approach to account for the ordered nature of the memory performance categories.

#### 2.7.2 ADNI study: application for CoxPH model

For survival modeling, we use data from the ADNI, a longitudinal study launched in 2003 to investigate multi-modal neuroimaging, biological markers, and clinical predictors of mild cognitive impairment (MCI) and AD progression. Our analyses use a recent data release, encompassing recruitment phases ADNI-1 (2004–2010), ADNI-GO (2009–2011), ADNI-2 (2011–2016), and the latest ADNI-3 (2016-present) cohort. ADNI involves various participant groups, including cognitively normal adults, individuals with significant memory concerns, early and late MCI participants, and those diagnosed with AD. For further information on data collection and processing, interested readers can consult the official website at http://www.adni-info.org. To construct SC matrices based on the MRI and DTI data from the ADNI study, regions of interest (ROIs) are first generated through anatomical parcellation of high-resolution T1-weighted MRI scans. The Lausanne parcellation scheme further subdivide these ROIs into smaller units, allowing for brain network construction at various scales (e.g., 83, 129, 234, 463, or 1,015 ROIs/nodes). The scale of connectivity that we put in our GenCPM pipeline is 83 ROIs. The T1-weighted MRI image is then registered to the low-resolution b0 image from DTI data to create a new set of ROIs in the DTI image space for structural network construction. The DTI data are then preprocessed to correct for motion and eddy current effects and fiber tracking was conducted using fiber assignment by continuous tracking (FACT). Nodes and edges are defined to create a weighted, undirected connectivity network represented by a matrix, where rows and columns correspond to the nodes and matrix elements indicate the weights of connections between nodes. The nodes are derived from the Lausanne parcellation and the edge weight between each pair of nodes is determined by the density of fibers connecting them.

The CoxPH model is applied to examine the effect of connectomes prior to the onset of *Aβ* pathology. In accordance with the literature on preclinical AD, we define the event of interest as the emergence of β-amyloid (*Aβ*) positivity, which indicates *Aβ* pathology accumulation during the asymptomatic stage of AD (Jack Jr et al., 2018). To determine *Aβ* positivity, we utilize the cortical-to-cerebellum standardized uptake value ratio (SUVR) measured via amyloid florbetapir ^18^F PET imaging and define *Aβ* positivity as having a global SUVR value above a specified cutoff of 1.4 (Vemuri et al., 2017). For our analysis, we include 1,217 participants from the ADNI cohorts who were initially event-free at the baseline visit. Among these subjects, the median follow-up time is 2.12 years, with initial follow-ups occurring every 6 months for the first 2 years and annually thereafter. During the follow-up period, 326 of the eligible participants developed *Aβ* pathology as defined, and 70 died without experiencing the event. As for non-imaging covariates, we take into account APOE4, gender (female vs. male) and handedness (left vs. right) in our model.

## 3 Results

### 3.1 Predictive performance

**Figure 3:**
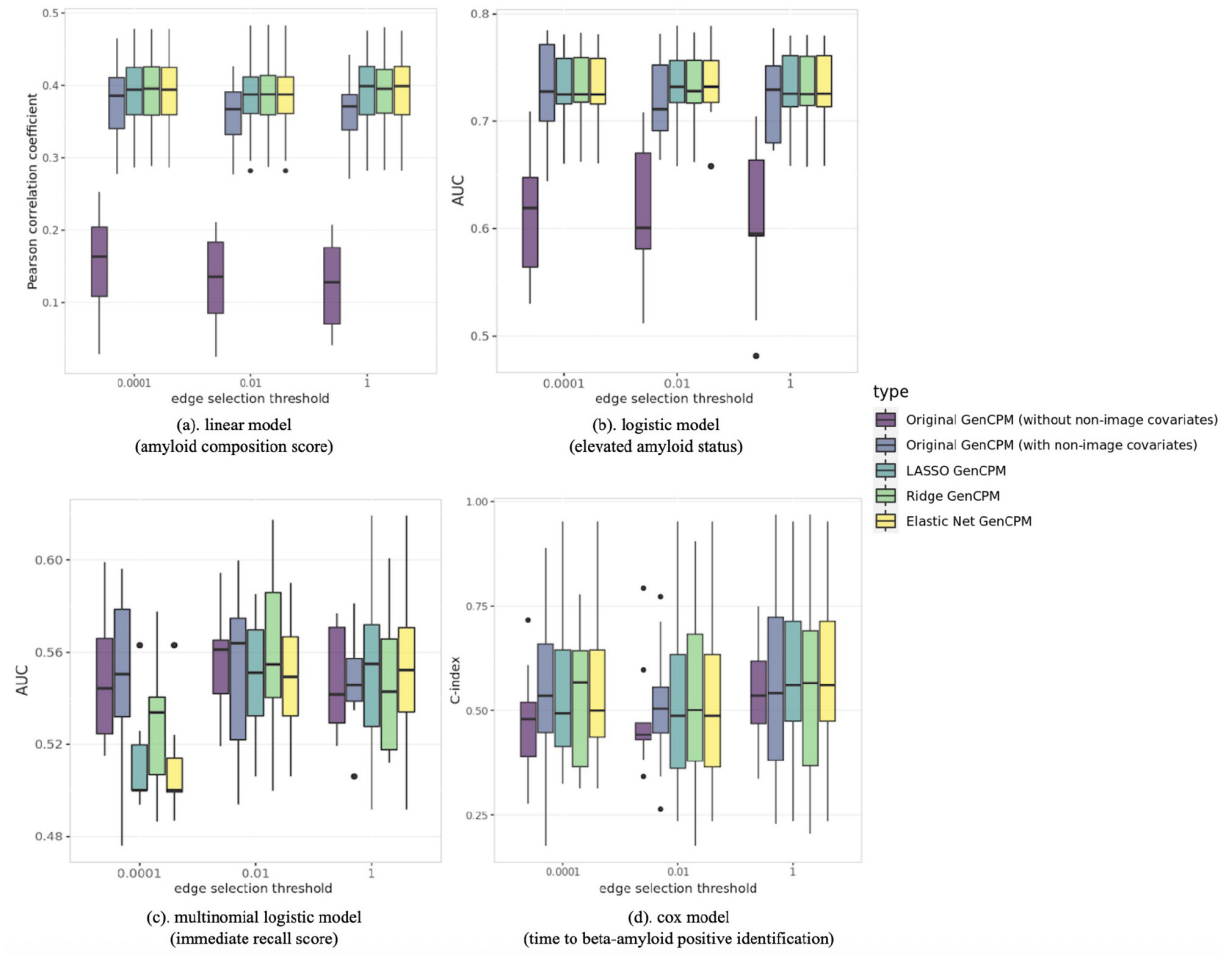
Predictive performance from 10-fold cross-validation of various GenCPM models in predicting amyloid composition score, elevated amyloid status, binned immediate recall score, and time to *Aβ* positive identification at different edge selection thresholds.

Figure 3 presents the boxplots demonstrating the performance of different generalized CPM models in predicting amyloid composition score, elevated amyloid status, binned immediate recall score, and time to *Aβ* positive identification. The models compared include GenCPM models both with and without non-imaging covariates as well as GenCPM models with LASSO, ridge, and elastic net penalty. The comparison also varies across different edge selection thresholds at 0.0001, 0.01, and 1. Specifically, when the threshold is specified as 1, no edge is preselected, and all edges are used for model fitting.

In the linear model (Figure 3a), assessing the amyloid composition score, the boxplots represent the distribution of the Pearson correlation coefficient. The Pearson correlation corresponding to GenCPM model introducing non-imaging covariates shows a marked difference from GenCPM without non-imaging covariates, suggesting that the additional non-imaging information may significantly influence the prediction of the amyloid composition score. The LASSO GenCPM, ridge GenCPM, and elastic net GenCPM, show performances that are similar to each other, but all offer a slight improvement over the original GenCPM approach without any penalization. In the original GenCPM without non-imaging covariates, the increase in threshold leads to a general pattern where the estimated correlation coefficient tends to decrease and the variability of the correlation tends to increase. This could indicate that there is a stronger and more consistent linear relationship between the predicted and observed behavioral measure when less edges are included at lower thresholds. However, the changes of estimated Pearson correlation and its variance across various thresholds are less pronounced in original GenCPM models with non-imaging covariates and penalized GenCPM models, which implies that the edge selection tends to play a more vital role when no non-imaging features are included.

The binary elevated amyloid status in the logistic model (Figure 3b) captures the same underlying clinical concept as the continuous amyloid composition score. This is reflected in the analogous patterns observed in Figures 3a, b, where (b) presents the distribution of AUC for elevated amyloid status prediction from 10-fold cross-validation. The correspondence in patterns across (a) and (b) underscores the conceptual similarity between the two amyloid measures. The boxplots in (b) also suggest that incorporating additional non-imaging features may significantly enhance the prediction of elevated amyloid status. The advanced regularized techniques consistently exceed the original GenCPM model, regardless of the inclusion of non-imaging features. Although the median AUC values of penalized GenCPM are approximately the same as those of the original GenCPM, this superiority is evidenced by the narrower interquartile ranges observed for these penalization methods, indicating enhanced predictive accuracy and increased stability across varying model thresholds.

In Figure 3c, we examine the distribution of multinomial AUC from a multinomial model aimed at predicting the binned immediate recall score. Similarly, the original GenCPM model is expected to improve the model's prediction by adding more features beyond connectivity. However, the observed improvement in this model is not as pronounced as it is in the linear and logistic models. At the lowest edge selection threshold of 0.0001, the three regularized methods instead have inferior performance compared to the original GenCPM method. This could be attributed to the limited number of significant edges selected at such a low threshold, leading to potential overfitting issues. This problem does not appear as the threshold increases to 0.01 and 1, which indicates a threshold-dependent performance characteristic of our pipeline. Furthermore, the predictive stability of the multinomial model is not as robust as that of the linear or logistic model. This suggests that the immediate score, perhaps due to its inherent complexity or the nature of the data, presents more challenges for stable modeling, particularly at lower thresholds where overfitting potentially happens. In Figure 3d, which compares the performance of a CoxPH model among different settings, the event of interest is the emergence of β-amyloid (Aβ) positivity and the model performance is evaluated using C-index. The boxplots demonstrate that while the regularized methods—LASSO GenCPM, ridge GenCPM, and elastic net GenCPM, have improved performance compared to the original GenCPM approach without non-imaging features, their predictive accuracy aligns closely with that of the original GenCPM method that includes non-imaging features. Another characteristic revealed in (d) is the relative insensitivity of the CoxPH model predictive accuracy to the number of significant edges included, as indicated by the minor change in performance across different threshold levels.

### 3.2 Edge selection

**Figure 4:**
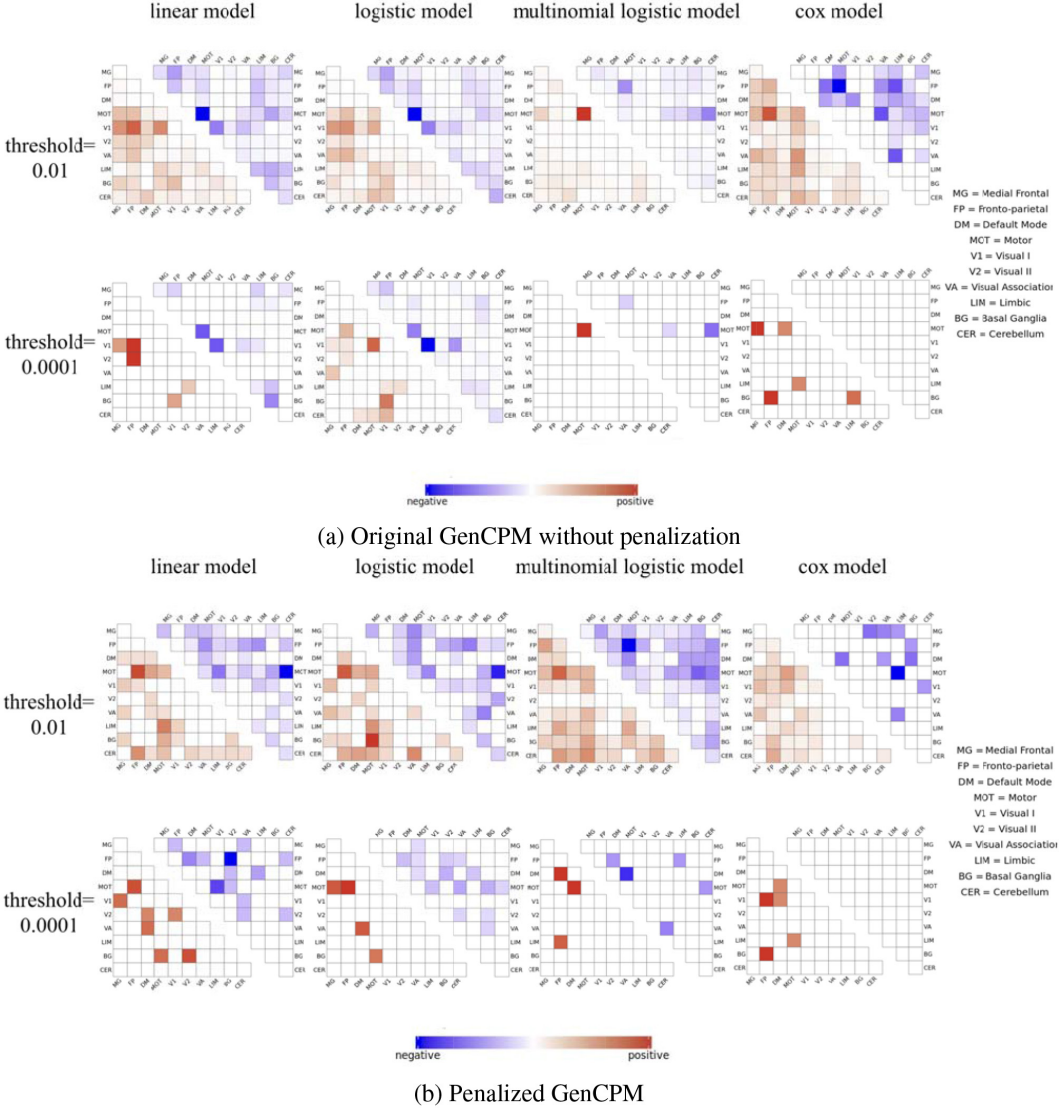
Most significant edges selected by **(a)** original GenCPM without penalization and **(b)** penalized GenCPM pipelines at different thresholds, separated by positive and negative correlation and summarized at the network level.

Figures 4a, b present positively and negatively significant edges correlated with various behavioral or clinical responses selected by different statistical models in the GenCPM with and without penalization. In both figures, the models include linear, logistic, multinomial, and CoxPH models, which respectively correspond to fit amyloid composition score, elevated amyloid status, binned immediate recall score, and time to the emergence of β-amyloid (*Aβ*) positivity. The matrices show the strength and direction of the associations between various brain functional networks and these four outcomes, which are indicated by the color gradient from negative (blue) to positive (red) correlations. Rows and columns of the heatmap represent 10 brain functional networks: MF, Medial Frontal; FP, Frontal-parietal; DM, Default Mode; MDT, Motor; V1, Visual I; V2, Visual II; VA, Visual Association; LIM, Limbic; BG, Basal Ganglia; CER, Cerebellum. Two significant thresholds (0.01 and 0.0001) are used to illustrate the robustness of the correlations across models.

It is intuitive and reasonable to see that the edge selection is sparser when the threshold decreases from 0.01 to 0.0001, demonstrated by less edges with color, no matter for which model and whether penalization applies or not. The more stringent threshold of 0.0001 filters out weaker associations that do not meet the strict criteria for significance. As a result, fewer edges are highlighted but with a deeper color indicating the strength of these associations. In Figure 4a, it is notable that the linear and logistic models show highly similar patterns of association due to their focus on amyloid composition score and elevated amyloid status that share closely related underlying information. Specifically, the intra-edges within MOT and V1 network show the most significantly negative correlation with both amyloid composition score and elevated amyloid status, and the pattern holds for both thresholds. At the selection threshold of 0.01, inter-edges between V1 and FP as well as V1 and MOT show the most significantly positive association with amyloid composition score while such positive association with elevated amyloid status identified by the logistic model is not as obvious as that by the linear model. The condition is slightly different when the threshold is 0.0001. In terms of significant edges correlated to amyloid composition score, positive edges mostly located in the network between V1 and FP as well as V2 and FP while the most significantly positive edge selected by the logistic model are between V1 and MOT. In the multinomial model, edges correlated to immediate recall score show a completely different pattern where the network between MOT and CER show the strongest negative association and a strong positive association is appeared in the network within MOT itself. As for the time to *Aβ* positivity, the correlated edges seem to be more spread out and not focused on a few individual networks. There is even no negative correlated edge selected when the threshold is too small at 0.0001.

Compared to Figure 4a, which demonstrates few significant associations and indicates that models may be conservative in identifying connections without penalization, there is a denser pattern of significant associations shown in Figure 4b including penalization. It suggests that penalization may help in revealing more associations that are potentially obscured by noise in the non-penalized models. The penalization appears to improve the sensitivity of the models by uncovering associations between connectivity and the outcomes of interest that may otherwise be difficult to detect, possibly due to weaker effect sizes or lower signal-to-noise ratios, making them more apparent when penalization is applied.

**Figure 5:**
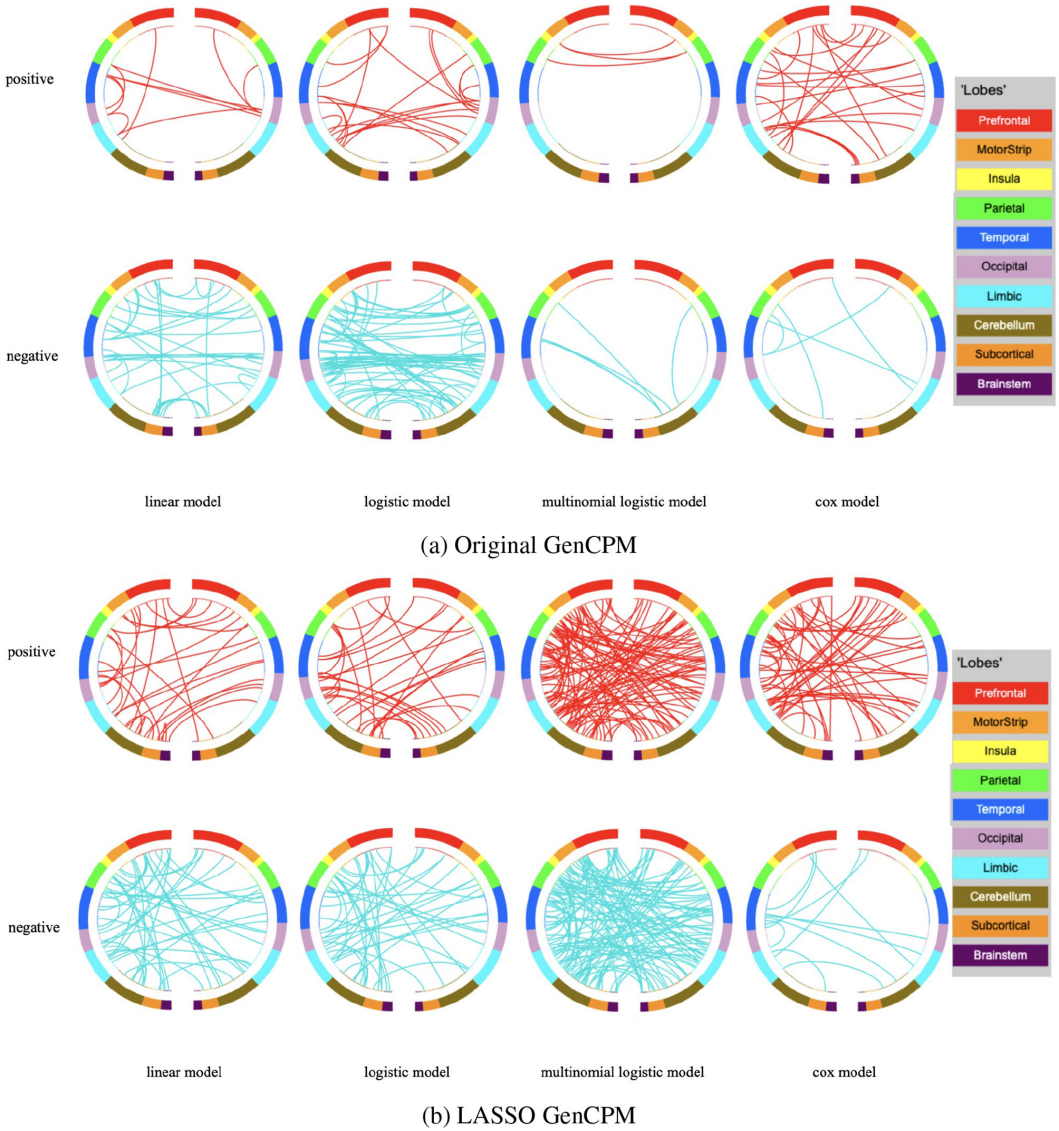
Node identification by **(a)** original GenCPM and **(b)** LASSO GenCPM.

One output of our GenCPM pipeline is the selected edges, including positive and negative ones. By importing them into BioImage Suite Web Connectivity Viewer https://bioimagesuiteweb.github.io/webapp/connviewer.html, we can have a better visualization of how edges intra- or inter-networks correlated with different behavioral and clinical responses. Node numbers are based on the Shen 268 atlas parcellation definition (Shen et al., 2013) and network are based on Yale network definition. Figures 5a, b illustrate the pattern of brain connectivity identified by the original GenCPM method (threshold = 0.0001) and by the LASSO GenCPM method (threshold = 0.01) respectively. Nodes that represent different regions of the brain are arranged in two half circles approximately reflecting brain anatomy from anterior (front) to posterior (back).

## 4 Discussion

In this study, we introduce GenCPM, a generalized connectome-based predictive modeling framework that extends traditional CPM by supporting a broader range of outcome types beyond continuous variables and by incorporating non-imaging covariates and confounders. These enhancements are particularly valuable in disease-focused connectomics research, where primary outcomes often include categorical diagnoses or time-to-event measures, and where non-imaging factors such as demographic, genetic, and clinical variables can substantially influence disease expression and progression. GenCPM offers two flexible analytical pipelines, allowing users to choose the optimal approach based on performance in their specific application. The toolbox is designed to be user-friendly and is implemented in R, a freely available and open-source platform, promoting accessibility without the need for proprietary software licenses. Through applications to two major Alzheimer's Disease studies, we demonstrate that GenCPM enables more comprehensive and interpretable modeling of disease mechanisms and cognitive variability. These capabilities make GenCPM a versatile and powerful tool for researchers and clinicians working to understand complex brain–behavior relationships in both health and disease.

To effectively handle the high dimensionality of connectivity data, GenCPM employs a two-stage feature selection strategy that balances interpretability and predictive performance. Marginal screening serves as an initial dimensionality reduction step to filter out features lacking a clear univariate association with the outcome; however, this approach does not account for dependencies or redundancies among predictors. This can lead to retaining correlated features together or excluding biologically meaningful variables whose information overlaps with others, an important limitation in neuroimaging where interpretability is critical. To address this limitation, the filtered features are subsequently analyzed using a generalized CPM with a user-selected penalization method (LASSO, ridge, or elastic net). These multivariate regularization techniques jointly consider correlations among predictors and encourage sparsity, resulting in a more stable and interpretable model. Together, our two-stage approach reduces noise while effectively managing inter-feature relationships, thereby enhancing the identification of key connectivity features that drive brain-behavior associations.

While GenCPM significantly advances the CPM framework, it remains linear in its modeling of covariates. Moving beyond linear models represents an important direction for future development. Mutual information-based feature selection offers a compelling alternative to traditional marginal screening, as it captures non-linear dependencies without assuming specific functional forms (Vergara and Estévez, 2014). However, it can be sensitive to noise and less interpretable in high-dimensional settings. Recent advances in explainable artificial intelligence (XAI) help address such challenges. While neural networks and ensemble methods (e.g., random forests and gradient boosting) are often viewed as “black boxes,” XAI techniques such as SHAP and LIME provide interpretable importance scores (Lundberg and Lee, 2017; Ribeiro et al., 2016). Integrating these tools in future versions of GenCPM may enable greater modeling flexibility while retaining transparency. Probabilistic graphical models, such as Bayesian networks, offer another promising direction. These models capture conditional dependencies and quantify uncertainty, valuable for analyzing interactions among imaging and non-imaging features (Koller and Friedman, 2009). Although they can be computationally intensive and sensitive to model specification, their ability to incorporate prior knowledge and handle missing data makes them appealing for integrative neuroimaging analysis. In summary, extending GenCPM to incorporate nonlinear frameworks could improve predictive performance and capture richer data structures. However, this requires balance with interpretability, which remains essential for scientific understanding and translational impact in neuroscience. Furthermore, as we continue to explore more suitable approaches for categorical outcomes, extending GenCPM to include ordinal logistic regression is a potential future improvement. This extension would better accommodate the ordered nature of certain outcome variables, such as memory scores, and provide more appropriate statistical modeling for these types of data.

Currently, GenCPM operates with the Shen268 functional atlas, which offers a practical balance between spatial resolution and computational efficiency. This fixed parcellation enables consistent edge definition and visualization across subjects but limits compatibility with alternative atlases and user-defined parcellation schemes. For example, the *heatmap.GenCPM* function currently supports only a fixed 10-network mapping based on the Shen268 atlas, which may not align with anatomical boundaries or white matter tracts when applied to structural connectivity data. This restriction can introduce ambiguity when interpreting selected edges in terms of network-level patterns. While GenCPM has been successfully applied to moderately large connectomes (e.g., 1,500–1,600 subjects), scalability remains a consideration as atlas resolution increases. Larger parcellations lead to quadratic growth in the number of edges, resulting in greater memory usage and longer runtimes during model fitting and cross-validation. Under the current implementation, typical GenCPM analyses with the Shen268 atlas complete within 1–4 h on a single CPU core with 30 GB of RAM, and peak memory usage remains under 20 GB. This makes GenCPM computationally feasible for standard datasets without requiring access to high-performance computing resources. To improve flexibility and scalability, future development will incorporate support for multiple parcellation schemes, including user-defined atlases. Planned enhancements include parallelized cross-validation and memory-efficient data structures to reduce runtime and accommodate higher-resolution connectomes. These improvements will broaden the applicability of GenCPM to a wider range of datasets and enhance the interpretability of network-level results across various brain mapping frameworks.

## Data Availability

Publicly available datasets were analyzed in this study. This data can be found here: data from the ADNI are publicly available at http://adni.loni.usc.edu; and data from the A4 study are available through the Laboratory of Neuro Imaging (LONI) Image and Data Archive (IDA) at https://ida.loni.usc.edu following approval of data use applications.
